# Novel mono‐ and multi‐strain probiotics supplementation modulates growth, intestinal microflora composition and haemato‐biochemical parameters in broiler chickens

**DOI:** 10.1002/vms3.709

**Published:** 2022-01-11

**Authors:** Rine Christopher Reuben, Shovon Lal Sarkar, Habiba Ibnat, Pravas Chandra Roy, Iqbal Kabir Jahid

**Affiliations:** ^1^ Department of Microbiology, Faculty of Biological Sciences and Technology Jashore University of Science and Technology Jashore Bangladesh; ^2^ German Centre for Integrative Biodiversity Research (iDiv) Halle‐Jena‐Leipzig, Leipzig University Leipzig Germany

**Keywords:** broiler, haemato‐biochemical parameter, intestinal microbiota, multi‐strain, probiotic

## Abstract

**Background:**

The reduction of antimicrobial usage in food‐producing animals necessitates the intense search for novel alternatives, including new probiotic strains with more effective properties in improving growth performance and curtailing diseases in animals.

**Objective:**

This study evaluated the effects of novel mono‐ and multi‐strain probiotics on the growth performance, intestinal microbiota and haemato‐biochemical parameters of broilers.

**Methods:**

A total of 160 one‐day‐old Cobb 500 broilers were divided into eight treatment groups with two replicates consisting of (1) basal diet (negative control), (2) basal diet with antibiotic, colistin sulphate, (3) basal diet with commercial probiotic, PROMAX® (positive control), (4) basal diet with *Pediococcus acidilactici* I5, (5) basal diet with *P. pentosaceus* I13, (6) basal diet with *Enterococcus faecium* C14, (7) basal diet with *Lactobacillus plantarum* C16 and (8) basal diet with the combination of all the four probiotic strains. Birds were kept for 35 days and through oral gavage, 1 ml of 108 study probiotic strains administered on days 3–6, 14 and 18.

**Results:**

Supplementation with *P. pentosaceus* I13, *L. plantarum* C16 or multi‐strain probiotics significantly (*p* < 0.05) improved the body weight gain and feed conversion ratio with decrease in feed intake and intestinal Enterobacteria counts. There was a significant (*p* < 0.05) increase in haemoglobin, mean corpuscular volume, total white blood cells, platelets counts and a lowered (*p* < 0.05) total cholesterol and glucose levels in multi‐strains probiotic supplemented birds.

**Conclusion:**

The supplementation with novel multi‐strain probiotics improved growth, intestinal health and haemato‐biochemical parameters in broilers and could be used as suitable antibiotic alternatives.

## Graphical abstract

This study evaluated the effect of novel probiotics supplementation on the growth performance, intestinal microbiota, and haemato‐biochemical parameters of broiler chickens. Birds were supplemented with mono and multi‐strains probiotics through oral gavage. The multi strain probiotics significantly improved growth performance, reduced intestinal Enterobacterial counts, increased haemoglobin, WBC and platelet counts, as well as lowering total cholesterol and glucose level in broilers.

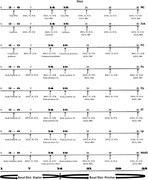

## 1 INTRODUCTION

With the total ban and restrictions on the use of antibiotics in animal production and poultry industry, probiotics have been widely and increasingly accepted as suitable, natural and safe alternatives to antibiotics (Olnood et al., 2015a). These beneficial microbial strains collectively referred to as probiotics are known to be “live microorganisms that when administered in adequate amounts, confer a health benefit on the host” (Hill et al., 2014). Probiotics confer multiple nutritional and health benefits, including improving animals’ performance and feed utilization (Mahmood et al., 2014), enhancing gut microflora (Olnood et al., 2015a), immune modulation (Salim et al., 2013), competitive pathogens exclusion (Rocha et al., 2012), enterotoxins neutralization (Rahimi, 2009), meat quality (Zhang & Kim, 2014), lowering cholesterol level (Ashayerizadeh et al., 2011), and reducing morbidity and mortality rates (Hatab et al., 2016; Salim et al., 2013), when administered in adequate amounts.

In the poultry industry, different probiotic strains belonging to *Bifidobacterium* spp., *Lactobacillus* spp., *Enterococcus* spp., *Bacillus* spp., *Streptococcus* spp., *Candida* spp., *Saccharomyces* spp. and *Aspergillus* spp. have been successfully applied and reported to improve poultry's performance (Chen et al., 2005; Chichowski et al., 2007; Liu et al., 2012). Although previous studies concentrated on mono‐strain probiotics, the application of multi‐strain probiotics is gradually now reported by many researchers with mixed outcomes (Abdel‐Latif et al., 2018; Olnood et al., 2015a). In a recent study, dietary supplementation with multi‐strains probiotic consisting of *Lactobacillus fermentum, L. plantarum, Enterococcus faecium, Pediococcus acidilactici* and *Saccharomyces cerevisiae* significantly improved growth performance, some haemato‐biochemical parameters as well as beneficially modulating gut microflora and also ameliorating *Pasteurella multocida* infection in broilers (Reuben et al., 2021). Similarly, Fesseha et al. (2021), Ramlucken et al. (2020), Kazemi et al. (2019) and Olnood et al (2015a) separately reported the effects of multi‐strain probiotics supplementation on growth performance, gut microbiome development and diversity, intestinal morphology, lipid oxidation and pathogens control in broiler chickens.

Some studies have previously reported discordant findings on the influence of probiotics supplementation on chicken haemoglobin, packed cell volume (PCV), total counts of red blood cells (RBCs), white blood cells (WBCs) and platelets, erythrocytes sedimentation rate (ESR), monocytes, glucose level, total cholesterol, total proteins, triglycerides and other blood parameters (Alkhalf, Alhaj & Al‐homidan, 2010; Abdel‐Hafeez et al., 2017; Deraz, 2018 Hussein, 2014), the direct influence exhibited by mono‐ and/or multi‐strains probiotic supplementation on haemato‐biochemical parameters of poultry has not been clearly elucidated.

In spite of the soaring acceptability of probiotics application in animal production, their functionality and efficacy in the field are highly inconsistent. This is partly because most commercial probiotics fall short of the basic standard and quality in both microbial viability and composition (Fasoli et al., 2003). The lack of generally acceptable administration dose in the field and the great variability in feed composition (Zhang et al., 2013) also affects the consistency of field reports. Furthermore, most manufacturers lack the patience to conduct in‐depth studies and in vivo trials for optimal efficacy of each probiotic strain before commercializing, as most industries are after profits maximization with minimal expense (Reuben et al., 2019).

The probiotic strains used in this study were isolated from indigenous raw milk and broilers, evaluated for probiotic properties, characterized molecularly and sequenced using 16S rRNA sequencing (Reuben et al., 2019; 2020). This experiment is the first to assess the field effectiveness of these newly identified probiotic strains with suitable in vitro probiotic properties. More so, most field experiments do not often evaluate the effect of mono‐ and multi‐strain probiotics simultaneously. Therefore, our study investigated the effects of individual and combined supplementation of novel strains of probiotics on growth performance, intestinal microflora and haemato‐biochemical parameters of broilers.

## 2 MATERIALS AND METHODS

### 2.1 The probiotic strains

Four potential probiotic strains previously isolated and identified as *P. acidilactici* I5 (chicken intestine), *P. pentosaceus* I13 (chicken intestine), *E. faecium* C14 (chicken crop) and *Lactobacillus plantarum* C16 (cow milk) were selected for this field trial. Each strain previously stored at –20°C in De Man, Rogosa, Sharpe (MRS) broth (Hi‐Media, M6411‐500G) with 40% glycerol was resuscitated by repeated culture in the MRS broth at 37°C for 24 hours and then harvested by centrifugation at 6000 x *g* for 5 minutes. Harvested cells were resuspended in phosphate buffered saline (PBS, pH 7.4) and vortexed for 10 minutes. The concentration of each probiotic strain supplemented in this study was 10^8^ cfu/ml. Also, commercial probiotic product PROMAX^®^ (Sanzyme Biologics (P) Ltd., Japan), which contained (per gram) 4.5 x 10^9^ cfu/g cells of *Bacillus subtilis*, *B. coagulans* and *Saccharomyces boulardii*, was used as positive control.

### 2.2 Experimental design and diet treatments

**FIGURE 1:**
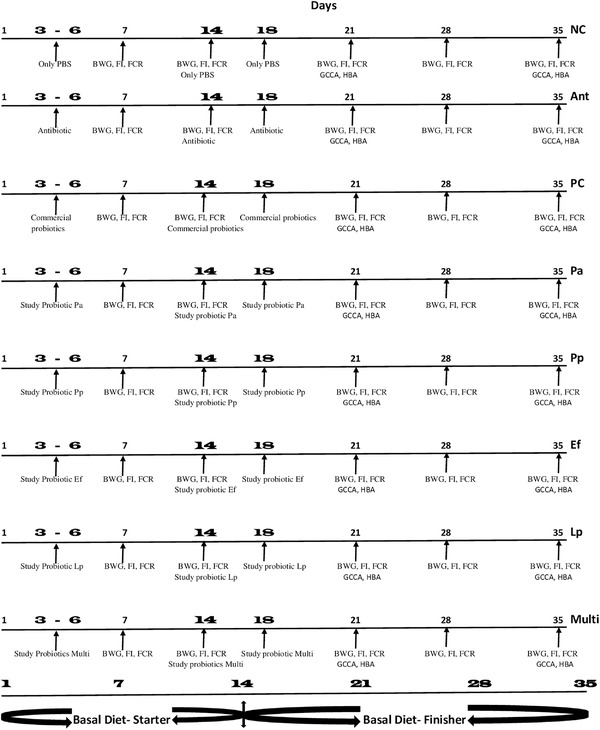
Schematic illustration of the experimental design. Birds were fed basal diet all through the experiment. Treatment groups included: NC, negative control; Ant, antibiotic supplemented; PC, positive control; Pa, supplemented with *Pediococcus acidilactici* I5; Pp, supplemented with *P. pentosaceus* I13; Ef, supplemented with *Enterococcus faecium* C14; Lp, supplemented with *Lactobacillus plantarum* C16 (Lp) and Multi, supplemented with Multi‐strains. Abbreviations: BWG, body weight gain; FCR, feed conversion ratio; FI, feed intake; GCCA, gut content and carcass analysis; HBA, haemato‐biochemical analysis; PBS, phosphate‐buffered saline

A total of 160 one‐day‐old Cobb 500 broiler mixed‐sex chicks were purchased from a commercial hatchery (Nourish farms, Dhaka, Bangladesh), weighed individually and randomly assigned to eight treatments with two replicates containing nine chicks each after they were allowed to acclimatize for 2 days. Birds in each treatment were housed in a floor pen containing sawdust litter. Twenty‐three hours of light was provided during the first week and then reduced to 18 hours throughout the 35 days of the experiment. Basal diets (starter and grower/finisher) designed in our laboratory were provided as pellets all through the trial and were based on wheat, soybean meal and corn (Table S1). The eight treatments adopted in this trial included: (1) negative control (NC), (2) antibiotic (colistin sulphate) supplemented (Ant), (3) positive control (PC) supplemented with commercial probiotic, (4) *P. acidilactici* I5 (Pa) supplemented, (5) *P. pentosaceus* I13 (Pp) supplemented, (6) *E. faecium* C14 (Ef) supplemented, (7) *L. plantarum* C16 (Lp) supplemented and (8) multi‐strains (Multi) supplemented group. In all the treatments, feed and water were provided ad libitum. The treatment procedures adopted for this study are shown in Figure 1.

### 2.3 Sample collection and processing

The individual weight of all chickens was measured before grouping them into respective treatment pens. Individual bird and leftover feed from each treatment were weekly weighed and the feed intake (FI) and body weight gain (BWG) recorded. Also, feed conversion ratio (FCR; feed intake/weight gain) and mortality (when it occurred) for each treatment were also calculated (Gao et al., 2008; Olnood et al., 2015a).

On days 21 and 35 of the experiment, two birds from each pen were selected at random and sacrificed by cervical dislocation after exposing them to overdose of isoflurane anesthesia. Visceral organs of each of the sacrificed bird were carefully removed and weighed after opening the abdominal cavity. After emptying the contents into sterile plastic containers, the weights of gizzard, ileum and caecum were recorded. Also, the weights of heart, liver, bursa, spleen, thigh, drumstick, breast, wing and dressing were recorded and expressed as the percentage of the body weight (Zhang et al., 2013).

### 2.4 Enumeration of culturable intestinal bacteria

For each bird sacrificed, fresh gizzard, ileum and caecum digesta were immediately collected within 1 hour for microbial enumeration. Using 0.85% normal saline solution, the fresh digesta samples were serially diluted for the enumeration of total aerobes, Enterobacteria (coliforms and lactose negative Enterobacteria) and lactic acid bacteria by conventional microbiological techniques using selective media, including nutrient agar, MacConkey agar and MRS agar, respectively (Engberg et al., 2004), and results were expressed as Log_10_ cfu/g.

### 2.5 Determination of digesta pH

Exactly 1 g of fresh digesta samples from gizzard, ileum and caecum of each sacrificed bird on days 21 and 35 were transferred into 9 ml of distilled water in 15 ml tubes and measured the pH using the standard procedures described elsewhere using glass electrode (HANNA Instruments, Inc., Woonsocket, RI, USA) (Kumar et al., 2018).

### 2.6 Haemato‐biochemical parameters

Complete blood counts and lipid profile determining the haemato‐biochemical parameters were carried out. Approximately 4 ml of blood samples from the birds sacrificed in each treatment were collected from the jugular vein into plane tubes (for biochemical analyses) and anticoagulant tubes (for haematological analysis) on days 21 and 35 of the trial. Haematological assays were conducted using automatic SYSAM‐XN‐1000, XN‐550 AL Random Access Haematology Machine (SYSMEX CORPORATION, Japan) and checked manually, while the biochemical analyses were carried out by Siemens Dimension RxL/Max/Vitros350 Random Access Chemistry Analyzer (Siemens Healthcare Diagnostics Inc, Tarrytown, NY, USA) after obtaining the serum through centrifugation. The average of results obtained from the haemato‐biochemical analyses per treatment were determined.

### 2.7 Statistical analysis

Data iwere collected iand analysed iby ianalysis iof ivariance ias ia icompletely irandomized idesign iusing ithe iGLM iprocedure as described by iGraphPad iPrism iversion i5.0 ifor iWindows i(GraphPad iSoftware, iSan iDiego, iCA, iUSA) and SAS software (version 9.4, SAS Institute Inc., Cary, NC, USA). Viable counts of the gizzard, ileum and caecum contents were subjected to logarithmic conversion (Log_10_) before statistical analysis. All the results iwere ipresented ias imeans of two independent experiments, iand differences ibetween itreatment igroups iwere determined using the Duncan's iimultiple iirange itest. Probability ivalue iless ithan i0.05 i(*p* < 0.05) iwas iconsidered as significant.

## 3 RESULTS

### 3.1 Growth performance

We prepared the basal diet for the study devoid of antibiotics. Also, the basal diet used in this study supported the general performance of the birds during the period of the experiment. The required composition of ingredients and calculated nutrients used in the basal diet during this study was arrived at after repeated pilot study involving 80 broilers (data not shown). The effects of dietary supplementation of mono‐ and multi‐strain probiotics on the BWG, FCR and FI of broiler chickens as obtained from this study are shown in Table 1. No significant differences (*p* > 0.05) were recorded in the BWG between the treatment groups with the NC and PC from days 1 to 14 of the experiment. At 21 days of age, the mean BWG of the mono‐strain probiotic groups Pp (860.17±13.91 g), Lp (915.60±13.91 g) and the Multi‐strain probiotics group, Multi (934.33±13.91 g) was significantly higher (*p* < 0.05) when compared with the NC (818.44±13.91 g) and PC (842.03±13.91 g) and the Ant (844.72±13.91 g) groups. This positive probiotic effect continued within the same groups of study probiotics with significantly higher (*p* < 0.05) BWG till day 35 of the experiment. Probiotics supplementation did not cause any significant increase in the FI during from days 1 to 7 of the experiment (*p* > 0.05). However, while this study recorded a significantly higher FI among all the probiotic supplemented groups on day 14 of the experiment, there was only an increased (*p* < 0.05) FI only in the Multi group on day 21, when compared with the NC. Conversely, there was a significant decrease (*p* < 0.05) in FI among birds in the probiotic supplemented groups when compared with the NC from day 28 until the end of the experiment. Furthermore, there were no significant differences (*p* > 0.05) recorded from this study in the means of FCR between study probiotics and the control groups on day 7. Nevertheless, at days 14, 21, 28 and 35 of age, this study recorded significant differences (*p* < 0.05) between the FCR of study probiotics and the NC. Although the NC and Ant groups showed higher FCR throughout the study period, study probiotic groups Lp and Multi showed the least FCR than the three other study probiotic‐treated groups, Pa, Pp and Ef as well as the NC and PC, respectively. Also, 5.56% and 6.25% mortality were recorded in the NC and Ant groups, respectively, on days 4 and 29 during the period of the experiment. Therefore, the percentage survivability at the end of the experiment was 94.40%, 93.75% and 100.00% for NC, Ant and other treatment groups, respectively. Although the survivability of birds supplemented with probiotics was 100.00% all through the experiment, birds in Pp, Lp and Multi treatment groups showed better performance than birds in other treatment groups (Table 2).

**TABLE 1:** Effects of mono‐ and multi‐strain probiotic strains on growth performance of broilers

Growth performance	Treatment									
	NC	Ant	PC	Pa	Pp	Ef	Lp	Multi	SEM	*p*‐Value
BW 1 (g)	50.11^a^	50.28^a^	50.72^a^	51.22^a^	51.50^a^	50.22^a^	51.94^a^	51.23^a^	0.24	0.862
BW 7 (g)	230.06^a^	228.89^a^	237.22^a^	229.50^a^	239.11^a^	238.67^a^	241.56^a^	237.00^a^	3.64	0.984
BWG (g)	179.95^a^	178.61^a^	189.28^a^	178.28^a^	187.61^a^	188.44^a^	189.61^a^	185.77^a^	3.57	0.999
FI (g)	146.22^a^	150.00^a^	175.05^a^	171.78^a^	163.56^a^	164.89^a^	177.50^a^	165.61^a^	3.96	0.484
FCR	0.813^a^	0.840^a^	0.925^a^	0.964^a^	0.879^a^	0.875^a^	0.936^a^	0.891^a^	0.010	0.324
BW 14 (g)	416.22^a^	405.00^a^	434.22^a^	404.00^a^	434.39^a^	455.61^a^	474.00^ab^	485.50^ab^	10.86	0.041
BWG (g)	366.11^a^	354.72^a^	385.50^a^	352.78^a^	382.89^a^	405.39^a^	422.06^a^	432.22^a^	10.59	0.068
FI (g)	346.67^g^	366.22^e^	370.72^b^	366.67^a^	348.61^d^	359.94^c^	390.72^d^	392.67^f^	6.02	0.512
FCR	0.947^d^	1.032^b^	0.962^c^	1.039^a^	0.910^f^	0.888 g	0.926^e^	0.908^e^	0.02	0.414
BW 21 (g)	868.56^a^	895.00^a^	893.06^a^	906.56^a^	911.67^ab^	899.11^a^	967.56^ac^	985.61^ac^	14.09	0.014
BWG (g)	818.44^c^	844.72^c^	842.33^c^	855.33^c^	860.17^b^	848.89^c^	915.60^a^	934.33^a^	13.91	0.004
FI (g)	706.83^b^	690.50^b^	716.61^b^	702.33^c^	701.56^b^	687.28^b^	711.67^b^	749.38^a^	6.83	0.198
FCR	0.864^a^	0.817^dc^	0.851^b^	0.821^c^	0.816^d^	0.810^e^	0.777 g	0.802^f^	0.01	0.023
BW 28 (g)	1337.75^a^	1339.13^a^	1349.44^a^	1357.50^a^	1394.00^c^	1346.38^a^	1382.38^c^	1447.13^bc^	13.23	0.041
BWG (g)	1287.69^c^	1289.06^c^	1298.44^c^	1306.13^c^	1342.31^b^	1295.75^c^	1330.31^b^	1395.25^a^	13.01	0.013
FI (g)	1073.56^a^	966.94^d^	935.50^e^	900.45^h^	894.94^b^	861.69 g	849.38^f^	915.88^c^	25.09	0.020
FCR	0.834^a^	0.750^b^	0.720^c^	0.689^d^	0.667^e^	0.665^e^	0.638 g	0.656^f^	0.02	0.017
BW 35 (g)	1795.06^a^	1836.40^a^	1882.44^a^	1897.73^a^	1913.19^ab^	1865.81^a^	2014.06^ac^	2052.50^ab^	30.64	0.014
BWG (g)	1745.00^d^	1786.27^d^	1831.43^dc^	1846.47^dc^	1861.50^c^	1815.19^dc^	1962.00^b^	2000.63^a^	30.39	0.011
FI (g)	1401.25^a^	1261.27^b^	1146.19^f^	1154.00 g	1129.00^e^	1211.38^c^	1186.42 g	1211.31^d^	30.90	0.001
FCR	0.803^a^	0.706^b^	0.626^d^	0.625^d^	0.607^e^	0.667^c^	0.605^f^	0.609^e^	0.02	0.021
Mortality	5.56	6.25	0.00	0.00	0.00	0.00	0.00	0.00	–	–

Note: Values are means of two replicates and standard errors of means. Within each variable, values with the same superscript letter are not significantly different according to Duncan's multiple range test (*p* > 0.05).

Abbreviations: Ant, antibiotic supplemented; Ef, supplemented with *E. faecium* C14; Lp, supplemented with *L. plantarum* C16 (Lp); Multi, supplemented with Multi‐strains; NC, negative control; Pa, supplemented with *P. acidilactici* I5; PC, positive control; Pp, supplemented with *P. pentosaceus* I13.

**TABLE 2:** Effects of mono‐ and multi‐strain probiotics supplementation on haematological parameters of broilers

	Treatment									
Parameter	NC	Ant	PC	Pa	Pp	Ef	Lp	Multi	SEM	*p*‐Value
**Day 21**										
RBC										
Total RBC (mil/Cmm)	2.41^a^	2.35^a^	2.37^a^	2.335^a^	2.54^a^	2.61^a^	2.67^a^	2.78^a^	0.09	0.516
Haemoglobin (g/dl)	6.75^b^	6.60^b^	6.85^b^	7.10^b^	6.41^b^	8.10^a^	8.05^a^	8.00^a^	0.16	0.029
ESR (mm/1 h)	2.00^a^	2.50^a^	1.50^a^	2.50^a^	2.00^a^	2.50^a^	2.50^a^	2.00^a^	0.13	0.623
PCV (%)	31.2^ab^	28.6^b^	31.7^ab^	32.1^ab^	31.85^ab^	31.80^ab^	33.10^a^	32.70^a^	0.48	0.021
MCV (fl)	142.45^a^	136.5^a^	139.4^a^	137.55^a^	133.25^a^	140.55^a^	129.00^a^	134.85^a^	1.53	0.733
MCH (pg)	30.35^a^	31.50^a^	29.70^a^	30.45^a^	28.55^a^	31.50^a^	31.40^a^	30.30^a^	0.36	0.836
MCHC (g/dl)	21.30^a^	23.05^a^	21.30^a^	22.15^a^	21.45^a^	22.35^a^	24.35^a^	22.45^a^	0.37	0.540
RDW (%)	12.55^e^	10.85^e^	12.30^e^	12.40^e^	13.10^e^	10.60^e^	10.35^e^	10.45^e^	0.39	0.218
WBC										
Total WBC (C/mm)	180,210^d^	164,855^d^	198,385^d^	316,700^d^	282,810^d^	283,630^d^	295,510^d^	280,235^d^	20,884.64	0.634
Neutrophils (%)	7.50^c^	4.50^c^	15.00^c^	4.00^c^	6.50^c^	12.50^c^	2.50^c^	11.50^c^	1.60	0.118
Lymphocytes (%)	90.50^e^	87.00^e^	92.00^e^	95.00^e^	91.00^e^	86.00^e^	97.00^e^	96.00^e^	1.43	0.489
Monocytes (%)	1.00^ae^	2.50^ae^	0.50^ae^	1.00^ae^	1.00^ae^	0.00^ae^	0.50^ae^	1.50^ae^	0.27	0.321
Basophiles (%)	1.50^ae^	0.50^ae^	3.00^ae^	0.00^ae^	1.50^ae^	1.50^ae^	0.00^ae^	2.00^ae^	0.37	0.267
Platelets										
Total platelet count (C/mm)	4000^ab^	4500^ab^	4000^ab^	5000^ab^	6000^a^	3000^ab^	5500^ab^	2500^b^	421.92	0.001
MPV (fl)	8.75^a^	9.65^a^	11.20^a^	10.40^a^	11.45^a^	9.65^a^	10.05^a^	10.40^a^	0.31	0.340
**Day 35**										
RBC										
Total RBC (mil/Cmm)	2.240^f^	2.95^f^	2.47^f^	2.38^f^	2.525^f^	2.63^f^	2.73^f^	2.89^f^	0.09	0.579
Haemoglobin (g/dl)	6.65^c^	7.65^abc^	7.00^c^	8.55^ab^	7.70^abc^	7.10^c^	7.45^bc^	8.85^a^	0.12	0.016
ESR (mm/1 h)	3.50^bcd^	2.00^e^	3.00^cde^	2.50^de^	4.5^ab^	4.00^abc^	3.5^bcd^	5.00^a^	0.35	0.001
PCV (%)	30.35^a^	33.75^a^	29.35^a^	34.05^a^	33.25^a^	30.45^a^	32.05^a^	34.00^a^	0.67	0.246
MCV (fl)	154.45^a^	146.3^ab^	132.4^b^	142.85^ab^	137.15^ab^	130.6^b^	132.8^b^	131.4^b^	3.04	0.028
MCH (pg)	31.85^a^	31.25^a^	31.70^a^	32.80^a^	31.80^a^	30.45^a^	30.85^a^	29.90^a^	0.32	0.402
MCHC (g/dl)	20.70^a^	21.35^a^	23.9^a^	22.95^a^	23.20^a^	23.40^a^	23.25^a^	22.80^a^	0.39	0.093
RDW (%)	10.90^ab^	11.30^ab^	10.95^ab^	9.85^ab^	9.50^ab^	9.85^ab^	8.90^b^	9.70^ab^	0.3	0.541
WBC										
Total WBC (C/mm)	97,620^b^	133,080^ab^	213,670^ab^	275,810^a^	275,810^ab^	207,880^ab^	281,675^a^	260,335^a^	145,76.7	0.031
Neutrophils (%)	38.00^ac^	40.50^ac^	16.00^ac^	19.00^ac^	25.50^ac^	23.50^ac^	31.00^ac^	34.00^ac^	3.14	0.344
Lymphocytes (%)	61.00^a^	57.00^a^	83.50^a^	80.00^a^	73.00^a^	75.50^a^	68.00^a^	70.50^a^	3.18	0.395
Monocytes (%)	1.00^ae^	2.00^ae^	0.00^ae^	0.00^ae^	2.00^ae^	1.00^ae^	0.50^ae^	1.00^ae^	0.27	0.461
Basophiles (%)	0.00^ab^	0.50^ab^	0.00^ab^	0.50^ab^	0.00^ab^	0.00^ab^	0.00^ab^	0.00^ab^	0.08	0.836
Platelets										
Total platelet count (C/mm)	4800^b^	7850^ab^	18,750^a^	7100^b^	8100^ab^	9000^ab^	6500^b^	8200^ab^	1469.75	0.039
MPV (fl)	9.35^a^	9.55^a^	10.30^a^	10.70^a^	9.40^a^	9.90^a^	9.05^a^	9.55^a^	0.19	0.465

Note: Values are the mean ± standard error of the mean of two replicates. Within each variable, values with the same superscript letter are not significantly different according to Duncan's multiple range test (*p* > 0.05).

Abbreviations: Ant, antibiotic supplemented; Ef, supplemented with *E. faecium* C14; ESR, erythrocyte sedimentation rate; Lp, supplemented with *L. plantarum* C16 (Lp); MCH, mean corpuscular haemoglobin; MCHC, mean corpuscular haemoglobin concentration; MPV, mean platelets volume; Multi, supplemented with Multi‐strains; MVC, mean corpuscular volume; NC, negative control; Pa, supplemented with *P. acidilactici* I5; PC, positive control; Pp, supplemented with *P. pentosaceus* I13; RDW, RBC distribution width.

### 3.2 Carcass and visceral organs weight

**FIGURE 2:**
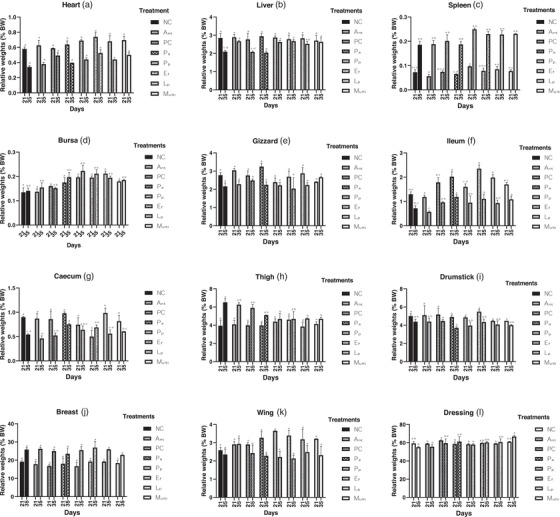
Relative weights (% BW) of organs from broilers supplemented with mono‐ and multi‐strain probiotics during 35 days trial. Note: Values are the mean ± standard error of the mean of two replicates. Within each variable, values with the same letter are not significantly different according to Duncan's multiple range test (*p* > 0.05). Abbreviations: Ant, antibiotic supplemented; Ef, supplemented with *E. faecium* C14; Lp, supplemented with *L. plantarum* C16 (Lp); Multi, supplemented with Multi‐strains; NC, negative control; Pa, supplemented with *P. acidilactici* I5; PC, positive control; Pp, supplemented with *P. pentosaceus* I13

The supplementation of study probiotics positively affected (*p* < 0.05) the relative weights of the spleen in Pp group; ileum in PC, Pa, Ef, Lp and Multi; and also a numerical increase (*p* > 0.05) in the relative weight of dressing in all the treatment groups when compared with the NC on day 21 of the experiment. Furthermore, on day 35 of the experiment, the relative weights of the liver in Pp, Ef, Lp and Multi groups; ileum in Pa, Ef and Multi groups; caecum in Pa and Ef groups; and dressing in Multi group were significantly higher (*p* < 0.05) than those of the NC and in some instances the PC and/or other study probiotic supplemented groups. The relative weights of heart, bursa, gizzard, duodenum, drumstick, breast, thigh and wing were not affected by the supplementation of probiotics during the entire period of the experiment (Figure 2).

### 3.3 Enumeration of bacterial population in intestinal digesta

**FIGURE 3:**
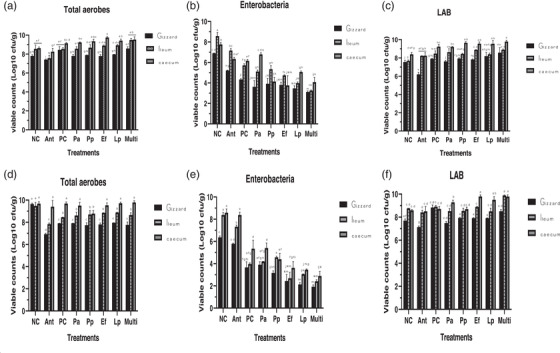
Bacterial counts (Log_10_ cfu/g) in the digesta of chickens supplemented with mono‐ and multi‐strains probiotic on day 21 (a–c) and day 35 (d–e). Note: Values are the mean ± standard error of mean of two replicates. Within each variable, values with the same superscript letter are not significantly different according to Duncan's multiple range test (*p* > 0.05). Abbreviations: Ant, antibiotic supplemented; Ef, supplemented with *E. faecium* C14; Lp, supplemented with *L. plantarum* C16 (Lp); Multi, supplemented with Multi‐strains; NC, negative control; Pa, supplemented with *P. acidilactici* I5; PC, positive control; Pp, supplemented with *P. pentosaceus* I13

The composition of the bacterial population in the intestinal digesta on days 21 and 35 of the experiment is shown in Figure 3. This study recorded a gradual increase in total aerobes and lactic acid bacteria (LAB) counts with decrease in Enterobacteria counts as the birds grow older. The viable counts of total aerobes in the gizzard, ileum and caecum ranged between 6.86 and 9.70, 7.77 and 9.26, and 9.00 and 9.68 Log_10_ cfu/g across the treatment groups on day 35 of the experiment. Probiotic supplementation grossly reduced (*p* < 0.05) the number of Enterobacteria in the gizzard, ileum and caeca with Lp and Multi groups showing the least counts both on days 21 and 35 of the experiment, respectively. However, LAB counts were significantly (*p* < 0.05) lower in caecum of birds in NC, PC and Ant groups when compared birds in all the treatment groups supplemented with the study probiotics either as mono‐ or multi‐strain.

### 3.4 Digesta pH

The evaluation of the digesta pH from the gizzard, ileum and caecum showed a gradual change from acidity to alkalinity from the upper (proximal) to the lower (distal) gut regions of the studied birds (Table S2). When compared to the NC and some other groups, the pH of the gizzard was significantly lowered in Multi group throughout the period of the experiment with pH values of 2.89 and 3.22 on days 21 and 35, respectively. This study did not record any significant effect (*p* > 0.05) on the pH of the ileum of the birds in all the treatment groups throughout the experiment. This study further revealed that the caeca of older birds (35 days of age) had increased pH values than younger birds (21 days of age). Also, in comparison with the NC, this study recorded an increase (*p* < 0.05) in the pH values of caecum in birds from the Ant, Pa, Pp, Ef and Multi groups.

### 3.5 Haemato‐biochemical parameters

With regard to the effects of probiotics supplementation on chicken haematological parameters, no significant changes (*p* > 0.05) were recorded on total RBC, mean corpuscular haemoglobin (MCH), mean corpuscular haemoglobin concentration (MCHC), Red Blood Cell distribution width (RDW), neutrophils, lymphocytes, monocytes, basophils and mean platelets volume (MPV) among all the treatment groups throughout the experiment (Table 2). While this study recorded a significant increase (*p* < 0.05) in both haemoglobin in birds in Ef, Lp and Multi groups and a numerical increase (*p* > 0.05) in PCV among birds in Pa, Pp, Ef, Lp and Multi groups on day 21, birds in Pa and Multi groups showed significantly higher haemoglobin values on day 35. Similarly, birds supplemented with the study probiotics showed numerical increase in PCV when compared with the NC on day 35 of the experiment. Although the total WBC count increases numerically in all the treatment groups in day 21, a significant increase (*p* > 0.05) was noticed among birds in Lp and Multi groups as birds in other probiotic supplemented groups showed no difference when compared to the NC on day 35. Furthermore, birds in only the Multi group showed significantly higher ESR values, while those in groups Pp and Ef had numerically higher values at the end of the experiment when compared with the NC, Ant and PC groups. At the end of the experiment, the total platelet count increased significantly (*p* < 0.05) only in birds in PC, nevertheless, birds in other treatment groups showed numerically higher counts when compared with the NC.

The serum biochemical parameters are shown in Table 3. Although this study recorded a significant decrease in total cholesterol in probiotic supplemented groups Lp and Multi when compared with the NC on day 21, there was numerical decrease in total cholesterol in Ant, PC and all the study probiotics supplemented groups with birds in the Multi group having the least value (49.00 mg/dl) on day 35 of the experiment. While birds in the Multi group had significantly (*p* < 0.05) reduced HDL cholesterol level, birds in other study probiotics groups showed reduced (*p* > 0.05) HDL cholesterol values on day 21 of the experiment. Similarly, this study also recorded a reduced (*p* > 0.05) LDL cholesterol level in birds supplemented with study probiotics when compared with the NC. High glucose levels of 14.21 and 14.41 mmol/L were recorded in birds from the NC group on days 21 and 35, respectively. Notably, birds in all the study probiotics treatment groups showed significantly lowered glucose levels when compared with the NC at the end of the experiment. This study did not show any significant difference in triglyceride, RISK (total cholesterol‐HDL ratio) and protein during the experiment. Although triglyceride values tended to decrease in probiotics supplemented groups, protein levels tended to increase in birds supplemented with probiotics throughout the trial.

**TABLE 3:** Effects of mono‐ and multi‐strain probiotics supplementation on serum biochemical parameters of broilers

	Treatment									
Parameters	NC	Ant	PC	Pa	Pp	Ef	Lp	Multi	SEM	*p*‐Value
**Day 21**										
Total cholesterol (mg/dl)	118.00^a^	113.50^a^	85.00^abc^	99.00^abc^	82.00^abc^	107.00^ab^	69.50^c^	88.50^bc^	2.26	0.039
HDL cholesterol (mg/dl)	97.00^a^	87.00^ba^	96.50^a^	94.50^a^	94.10^a^	96.50^a^	87.50^b^	91.00^a^	1.43	0.033
LDL cholesterol (mg/dl)	8.70^ab^	12.40^a^	10.30^a^	10.90^a^	6.80^ab^	5.40^ab^	1.95^b^	2.90^b^	1.35	0.022
Triglyceride (mg/dl)	123.50^a^	117.00^a^	119.00^a^	116.00^a^	111.50^a^	114.50^a^	108.00^a^	109.50^a^	1.82	0.344
RISK	1.17^a^	1.11^a^	1.15^a^	1.21^a^	1.17^a^	1.20 ^a^	1.14^a^	1.19^a^	0.01	0.718
Total protein (g/dl)	2.55^a^	2.72^a^	2.78^a^	2.73^a^	2.58^a^	2.91^a^	3.05^a^	2.82^a^	0.06	0.281
Glucose (mmol/L)	14.21^a^	13.41^ab^	13.09^b^	13.07^ab^	13.19^ab^	13.06^b^	12.69^bc^	12.1^c^	0.21	0.046
**Day 35**										
Total cholesterol (mg/dl)	98.50^a^	92.00^a^	71.00^a^	91.00^a^	93.00^a^	52.00^a^	94.50^a^	49.00^a^	7.09	0.285
HDL cholesterol (mg/dl)	62.00^ab^	82.00^a^	49.50^ab^	71.50^ab^	70.00^ab^	24.50^b^	74.00^ab^	47.50^ab^	6.6	0.034
LDL cholesterol (mg/dl)	20.10^a^	6.00^a^	11.55^a^	25.00^a^	8.43^a^	21.60^a^	24.50^a^	19.50^a^	2.61	0.483
Triglyceride (mg/dl)	75.50^a^	73.50^a^	124.50^a^	96.50^a^	157.50^a^	156.50^a^	101.50^a^	144.50^a^	12.16	0.719
RISK	2.48^cd^	1.11^cd^	1.53^cd^	1.27^cd^	1.33^cd^	5.19^cd^	1.28^cd^	3.46^cd^	0.51	0.601
Total protein (g/dl)	2.84^a^	2.90^a^	2.75^a^	3.02^a^	3.05^a^	3.08^a^	2.83^a^	2.89^a^	0.04	0.087
Glucose (mmol/L)	14.41^a^	12.91^ab^	9.32^c^	9.09^c^	9.10^c^	10.58^bc^	8.44^c^	9.23^c^	0.76	0.038

Note: Values are the mean ± standard error of the mean of two replicates. Within each variable, values with the same superscript letter are not significantly different according to Duncan's multiple range test (*p* > 0.05).

Abbreviations: Ant, antibiotic supplemented; Ef, supplemented with *E. faecium* C14; HDL, high‐density lipid; LDL, low‐density lipid; Lp, supplemented with *L. plantarum* C16 (Lp); Multi, supplemented with Multi‐strains; NC, negative control; Pa, supplemented with *P. acidilactici* I5; PC, positive control; Pp, supplemented with *P. pentosaceus* I13; RISK, total cholesterol‐HDL ratio.

## 4 DISCUSSION

Dietary supplementation of a mono‐ or multi‐strain probiotics in broilers has been reported to promote the general performance and health of broilers by modulating intestinal microbiome, improving digestion and enhancing immunomodulation (Yang et al., 2012). Findings from this study revealed that the supplementation of novel multi‐strain probiotic consisting of *P. acidilactici* I5, *P. pentosaceus* I13, *E. faecium* C14 and *L. plantarum* C16 through the oral gavage significantly improved the BWG and FCR from day 21 to 35 of the experiment, with decrease in FI (Table 1). The supplementation with individual probiotic strains, including *P. pentosaceus* and *L. plantarum*, also shows increase in BWG on day 21, 28 and 35, respectively. Although there was a significant increase in FI among birds supplemented with single strain of the study probiotic candidates on day 14 of the experiment, conversely on days 28 and 35 of the experiment, birds supplemented with the study single‐ and multi‐strain probiotics showed significantly reduced FI. In agreement with our findings, multiple independent research (Bostami et al., 2015; 2016; Zhang and Kim, 2014) reported that the dietary supplementation of multi‐strain probiotics improved the BWG and FCR of broilers significantly, while the FI remains unaffected. On the contrary, Hossain et al. (2015) and Balamuralikrishnan et al. (2017) reported increased BWG with no effects on FI and FCR after supplementing multi‐strain probiotics on broilers.

The significant effects on BWG due to the supplementation of single strain of probiotic, *P. pentosaceus* and *L. plantarum* and also FCR for all the single strain of study probiotics are in consonance with the reports of several authors (Cao et al., 2013; Huang et al., 2004; Jin et al., 2000). Conversely, several studies also found no or minimal effect of single‐strain probiotics on the growth performance of broilers (Huang et al., 2004; Olnood et al., 2015a). The magnitude of the improvement of broilers performance is dependent on the probiotic strain used as single or as combination couple with the conditions under which they are applied (Olnood et al., 2015b).

Based on the findings of this current study, multi‐strain probiotics supplementation can improve the growth performance of broilers better than mono‐strain probiotics. This could be attributed to the synergistic actions of the combined strains, which positively improves nutrients utilization, sugars fermentation, synthesis of enzymes, increases in the secretion of beneficial metabolites and enhances antagonism against pathogens in broilers gut (Chapman et al., 2011; Szymanowska‐Powałowska et al., 2014). Similarly, multi‐strain probiotics supplementation in broilers has been evidently revealed to be more efficacious than mono‐strain probiotics (Timmerman et al., 2004) and also, optimal health benefits are elicited by host‐specific probiotic strains (Timmerman et al., 2005). Furthermore, the increase in BWG and improved FCR as recorded from this study may be due to the ability of the probiotic strains to improve the efficiency of digestion and subsequent absorption of nutrient within the gastrointestinal tract (GIT) of broilers.

Our findings showed a reduction in the mortality rate of probiotic supplemented birds, which is similar to other findings (Abdel‐Latif et al., 2018; Cmiljanic et al., 2001).

One of the major indicators of the effect of probiotic supplementation on broilers is the changes in the relative weight of visceral organs and carcass. Although the current study reported inconsistencies in the effects of specific probiotic strains on the relative weight of organs, birds supplemented with *P. pentosaceus* showed significant increase in relative weight of spleen on day 21 of age (Figure 2). Also, there was an increase in the relative weight of liver among birds supplemented with *P. pentosaceus*, *E. faecium*, *L. plantarum* and multi‐strain probiotics when compared with the controls (NC and PC). The relative weight of both the ileum and caecum increased significantly only in birds supplemented with mono‐strains probiotic candidates, *P. acidilactici* and *E. faecium* as well as multi‐strains supplemented birds when compared with the NC and antibiotic supplemented groups (Figure 2). Although the inclusion of a mono‐strain probiotic (*B. subtilis*) increased the relative weight of spleen by 3.8% in broilers, the relative weights of liver and bursa of Fabricius were unaffected (Zhang et al., 2013). Probiotics effect on the weight of visceral organs and intestines of animals is inexplicit, and can also be determined by the nature and concentration of either the single strain or combination of strains used as probiotics. It has been reported that probiotics consistently influence the intestinal morphology and micro‐structure, which often increases the absorptive function of the ileum (Olnood et al., 2015b; van Dijk et al., 2002).

The current study demonstrates that the GIT microbiota of broilers can be significantly influenced by the supplementation of broilers with multi‐strain probiotics. Also, some mono‐strain probiotics as revealed from this study also have the ability to cause significant changes in the GIT of broilers. The population of Enterobacteria significantly reduced in the gizzard, ileum and caecum with the inclusion of the study probiotics when compared with the control. Although the gizzard had the least Enterobacteria count, the reduction of Enterobacteria tended to improve with increase in age of the birds, as the multi‐strain probiotics supplemented birds had the least counts, which is consistent with other findings (Lan et al., 2003; Teo & Tan, 2007; Van der et al., 2002; Zhang & Kim, 2014). Other reports using multi‐ (Priyankarage et al., 2003) and mono‐strain (Zhang et al., 2013) probiotics showed no changes in the microflora of broilers, which differs with the current finding.

Although the supplementation with multi‐strain probiotics as observed from this study increased the population of LAB in the gizzard, ileum and caecum contents, birds supplemented with single strain of *E. faecium* or *L. plantarum* also had high LAB counts in their ileum and caecum contents, respectively. However, the comparison of studies on the effect of mono‐ or multi‐strain probiotics on intestinal microflora is difficult because probiotics influence in the GIT depends on the nature and viability of strain(s) used, dosage, method of application, bird age, diet used, farm hygiene and other environmental factors (Patterson & Burkholder, 2003). The gradual increase in the pH from the proximal to the distal GIT regions of birds supplemented with probiotics as shown in this study corroborated with other reports (Olnood et al., 2015a; 2015b). Birds supplemented with multi‐strain probiotics as well as those given *E. faecium* or *L. plantarum* tended to show more acidity in their gizzard and ileum. The highly acidic gizzard environment as recorded in this study could be one of the major factors that reduced the population of total aerobes as well as other Enterobacteria (which are mostly pathogenic) from accessing the distal regions of the GIT, hence their lower counts.

Although this study recorded no significant differences in some haematological parameters examined, birds supplemented with probiotics showed numerical increase in total RBC, MCHC, MPV and lymphocyte counts. Contrary with our findings, Alkhalf et al. (2010) and Dimcho et al. (2005) reported no effect on haematological parameters, including PCV and haemoglobin concentration of birds supplemented with probiotics. In agreement with our findings, Cetin, Guclu and Cetin (2005) observed a statistical increase in ESR, haemoglobin concentration and haematocrit values in birds supplemented with probiotics. Also, the supplementation of either single strain of *L. lactis* and *L. plantarum* or their combination as multi‐strain probiotics significantly increases the total RBC counts and haemoglobin concentration (Deraz, 2018) as shown in the present study. Arising from our study, the significant increase in total WBC count, total platelet counts, haemoglobin, PCV and ESR in one or more of the single and/or multi‐strain probiotics supplemented birds agrees with literature reports. Significant increase in RBC and WBC counts and ESR concentration were observed by Paryad and Mahmoudi (2008) and Cetin et al. (2005) when mono‐ and multi‐strain probiotics (Deraz, 2018) were supplemented in birds. The dietary inclusion of probiotics positively influenced haematopoiesis, which among others increase the WBC counts, hence enhancing immune cells synthesis, which further protects the host against invading pathogens (LaFleur & LaFleur, 2008; Gaggıa et al., 2010).

The decrease in key biochemical parameters, including total cholesterol, HDL cholesterol and LDL cholesterol as reported in this study, is in consonance with the work of Arun et al. (2006), who reported a significant reduction in total cholesterol and triglycerides by dietary inclusion of 100 mg/kg diet of *L. sporogene* probiotic in broilers. In his work, Deraz (2018) reported a non‐significant decrease in both total cholesterol and triglyceride levels after the supplementation of two mono‐ and multi‐strain probiotics on broilers. Total cholesterol reduction in probiotic supplemented birds could be as a result of direct assimilation of cholesterol by bacterial cells (which causes reduction in the cholesterol absorption and synthesis in the GIT), 3‐hydroxy‐3‐methyl‐glutaryl‐CoA reductase inhibition and bile salt hydrolysis (Fukushima & Nakano, 1995; Mohan et al., 1996). Furthermore, triglyceride reduction in probiotic‐treated birds may be as a result of increased hydrolysis of bile salt, which causes inadequate lipid absorption in the small intestine (Alkhalf et al., 2010). Strains of *Lactobacillus* are known to show high hydrolytic activity on bile salt, which consequently leads to bile salts deconjugation within the GIT (Surono et al., 2003).

The significant decrease in glucose levels of birds supplemented with all the mono‐strain probiotic candidates as well as the multi‐strain supplemented birds (which have the least glucose level) agrees with the report of Al‐Kassie et al. (2008). It has been previously reported that the relationship existing between blood glucose levels and probiotic inclusion is dose dependent (Samanya & Yamauchi, 2002). Also, the addition of probiotics as recorded from this study had no significant effect on total protein when compared with the NC. Although triglyceride values tended to decrease in probiotics supplemented groups (in day 21), protein levels tended to increase in birds supplemented with probiotics as reported from the present report. This corroborated with the findings of Dimcho et al. (2005), Alkhalf et al. (2010) and Abdel‐Hafeez et al. (2017) who unanimously reported no effect on total protein concentration in chickens supplemented with probiotics.

## 5 CONCLUSIONS

Although findings from this study showed that supplementation with novel mono‐strain probiotics *P. pentosaceus* I13 or *L. plantarum* C16 could improve the growth performance of broilers, supplementation with multi‐strain probiotics has more beneficial effects in both the growth performance and haemato‐biochemical parameters. Furthermore, multi‐strain probiotics ability to grossly reduce the number of Enterobacteria while improving gut health is a major attribute of their positive effects in pathogens control in poultry. Future research would centre on the development of commercial probiotics with pathogens challenge and metagenomics analysis.

## CONFLICT OF INTEREST

The authors declare that the research was conducted in the absence of any commercial or financial relationships that could be construed as a potential conflict of interest.

## ETHICAL STATEMENT

The authors confirm that the ethical policies of the journal, as noted on the author guidelines, have been adhered to and the appropriate ethical review committee approval has been received. The field trial was approved by the Animal Care and Use Committee of the Faculty of Biological Science and Technology, Jashore University of Science and Technology, Jashore, Bangladesh (certification number: ERC/FBST/JUST/2019‐32). The authors confirm that they have followed EU standards for the protection of animals used for scientific purposes. The health status of birds in the field was routinely monitored by a veterinarian. The birds were kept under controlled environmental conditions in the animal house of Jashore University of Science and Technology, Jashore, Bangladesh, throughout the experimental period.

## AUTHOR CONRIBUTIONS

Rine Christopher Reuben: data curation; formal analysis; validation; and writing – original draft. Shovon Lal Sarkar: data curation; formal analysis; investigation; project administration; software; visualization; and writing – review and editing. Habiba Ibnat: formal analysis; methodology; resources; and visualization.

### PEER REVIEW

The peer review history for this article is available at https://publons.com/publon/10.1002/vms3.709

## Supporting information



Supporting Information

## Data Availability

All the data shown in the manuscript were obtained from the result of this experiment and readily available to the reader and the data is available to use by any similar types of research.

## References

[vms3709-bib-0001] Abdel‐Hafeez, H. M. , Elham, S. , Saleh, E. , Samar, S. T. , Ibrahim, M. I. Y. , & Asmaa, S. A. A. (2017). Effects of probiotic, prebiotic, and synbiotic with and without feed restriction on performance, hematological indices and carcass characteristics of broiler chickens. Asian–Australas Journal of Animal Science, 30, 672–682. 10.5713/ajas.16.053 PMC5411827 27620891

[vms3709-bib-0002] Abdel‐Latif, M. A. , Mohamed, E. A. , Ayman, A. S. , Islam, M. S. , Ahmed, R. E. , & Ramadan, S. S. (2018). Single and combined effects of *Clostridium butyricum* and *Saccharomyces cerevisiae* on growth indices, intestinal health, and immunity of broilers. Animals, 8, 184. 10.3390/ani8100184 PMC6210252 30347769

[vms3709-bib-0003] Al‐Kassie, G. , Al‐Jumaa, Y. , & Jameel, Y. (2008). Effect of probiotic (*Aspergillus niger*) and prebiotic (*Taraxacum officinale*) on blood picture and biochemical properties of broiler chicks. International Journal of Poultry Science, 7(12), 1182–1184.

[vms3709-bib-0004] Alkhalf, A. , Alhaj, M. , & Al‐homidan, I. (2010). Influence of probiotic supplementation on blood parameters and growth performance in broiler chickens. Saudi Journal of Biological Science, 17, 219–225. 10.1016/j.sjbs.2010.04.005 PMC3730717 23961081

[vms3709-bib-0005] Arun, K. P. , Rama, R. , Savaram, V. , Mantena, V. L. N. , & Raju, S. R. S. (2006). Dietary supplementation of *Lactobacillus sporogenes* on performance and serum biochemico‐lipid profile of broiler chickens. Journal of Poultry Science, 43, 235–240.

[vms3709-bib-0006] Ashayerizadeh, A. , Dabiri, N. , Mirzadeh, K. , & Ghorbani, M. (2011). Effect of dietary supplementation of probiotic and prebiotic on growth indices and serum biochemical parameters of broiler chickens. Journal of Cell and Animal Biology, 5, 152–156.

[vms3709-bib-0007] Balamuralikrishnan, B. , Lee, S. I. , & Kim, I. H. (2017). Dietary inclusion of different multi‐strain complex probiotics on performance in broilers. British Poultry Science, 58, 83–86. 10.1080/00071668.2016.1257112 27918205

[vms3709-bib-0008] Bostami, A. B. M. , Ahmed, S. T. , Mun, H. S. , Hong, S. B. , & Yang, C. J. (2016). Efficacy of Rhodopseudomonas containing multi‐microbe probiotic on growth performance, mortality and cecal microflora in broilers. African Journal of Microbiology Research, 10, 985–993.

[vms3709-bib-0009] Bostami, A. B. M. , Islam, R. M. M. , Ahmed, S. T. , Mun, H. S. , Hong, S. B. , & Yang, C. J. (2015). Effect of beneficial microorganisms on growth performance, mortality and intestinal microflora in broilers. Global Journal of Microbiology Research, 3, 126–133.

[vms3709-bib-0010] Cao, G. T. , Zeng, X. F. , Chen, A. G. , Zhou, L. , Zhang, L. , & Xiao, Y. P. (2013). Effects of a probiotic, *Enterococcus faecium*, on growth performance, intestinal morphology, immune response, and cecal microflora in broiler chickens challenged with *Escherichia coli* K88. Poultry Science, 92, 2949–2955. 10.3382/ps.2013-03366 24135599

[vms3709-bib-0011] Cetin, N. , Guclu, B. K. , & Cetin, E. (2005). The effects of probiotic and mannanoligosaccharide on some haematological and immunological parameters in Turkeys. Journal of Veterinary Medical. A, Physiology, Pathology, Clinical Medicine, 52, 263–267. 10.1111/j.1439-0442.2005.00736.x 16050905

[vms3709-bib-0012] Chapman, C. M. , Gibson, G. R. , & Rowland, I. (2011). Health benefits of probiotics: Are mixtures more effective than single strains? European Journal of Nutrition, 50, 1–17. 21229254 10.1007/s00394-010-0166-z

[vms3709-bib-0013] Chen, Y. J. , Son, K. S. , Min, B. J. , Cho, J. H. , Kwon, O. S. , & Kim, I. H. (2005). Effects of dietary probiotic on growth performance, nutrients digestibility, blood characteristics and fecal noxious gas content in growing pigs. Asian–Australas Journal of Animal Science, 18, 1464–1468.

[vms3709-bib-0014] Chichowski, M. , Croom, W. J , Edens, F. W. , MacBride, B. W. , Qiu, R. , & Chiang, C. C. (2007). Microarchitecture and spatial relationship between bacteria and ileal, cecal and colonic epithelium in chicks fed a direct‐fed microbial, PrimaLac, and Salinomycin. Poultry Science, 86, 1121–1132. 10.1093/ps/86.6.1121 17495082

[vms3709-bib-0015] Cmiljanic, R. , Lukic, M. , & Trenkovski, S. (2001). The effect of “Paciflor‐C” probiotic on gain, feed conversion and mortality of fattening chicks. Biotechnology and Animal Husbandry, 17, 33–38.

[vms3709-bib-0016] Deraz, S. F. (2018). Synergetic effects of multispecies probiotic supplementation on certain blood parameters and serum biochemical profile of broiler chickens. Journal of Animal Health and Production, 6, 27–34. 10.17582/journal.jahp/2018/6.1.27.34

[vms3709-bib-0017] Dimcho, D. , Svetlana, B. , Tsvetomira, S. , & Tatiana, V. (2005). Effect of feeding Lactina probiotic on performance, some blood parameters and caecal microflora of mule ducklings. Trakia Journal of Science, 3, 22–28.

[vms3709-bib-0018] Engberg, R. M. , Steenfeldt, S. , Hedemann, M. S. , & Jensen, B. B. (2004). The influence of whole wheat and xylanase on broiler performance and microbial composition and activity in the digestive tract. Poultry Science, 83, 925–938. 10.1093/ps/83.6.925 15206619

[vms3709-bib-0019] Fasoli, S. , Marzotto, M. , Rizzotti, L. , Rossi, F. , Dellaglio, F. , & Torriani, S. (2003). Bacterial composition of commercial probiotic products as evaluated by PCR‐DGGE analysis. International Journal of Food Microbiology, 82, 59–70. 12505460 10.1016/s0168-1605(02)00259-3

[vms3709-bib-0020] Fesseha, H. , Demlie, T. , Mathewos, M. , & Eshetu, E. (2021). Effect of *Lactobacillus* species probiotics on growth performance of dual‐purpose chicken. Veterinary Medicine: Research and Report, 12, 75–83. 10.2147/VMRR.S300881 PMC8039195 33854957

[vms3709-bib-0021] Fukushima, M. , & Nakano, M. (1995). The effect of probiotic on faecal and liver lipid classes in rats. British Journal of Nutrition, 73, 701–710. 10.1079/bjn19950074 7626589

[vms3709-bib-0022] Gaggıa, F. , Mattarelli, P. , & Biavati, B. (2010). Probiotics and prebiotics in animal feeding for safe food production. International Journal of Food Microbiology, 141, 15–28. 10.1016/j.ijfoodmicro.2010.02.031 20382438

[vms3709-bib-0023] Gao, J. , Zhang, H. J. , & Yu, S. H. (2008). Effects of yeast culture in broiler diets on performance and immunomodulatory functions. Poultry Science, 87, 1377–1384. 10.3382/ps.2007-00418 18577619

[vms3709-bib-0024] Hatab, M. H. , El sayed, M. A. , & Ibrahim, N. S. (2016). Effect of some biological supplementation on productive performance, physiological and immunological response of layer chicks. Journal of Radiation Research and Applied Sciences, 9, 185–192. 10.1016/j.jrras.2015.12.008

[vms3709-bib-0025] Hill, C. , Guarner, F. , Reid, G. , Gibson, G. R. , Merenstein, D. J. , & Pot, B. (2014). The International Scientific Association for Probiotics and Prebiotics consensus statement on the scope and appropriate use of the term probiotic. Nature Reviews Gastroenterology and Hepatology, 11, 506–514. 24912386 10.1038/nrgastro.2014.66

[vms3709-bib-0026] Hossain, M. M. , Begum, M. , & Kim, I. H. (2015). Effect of *Bacillus subtilis*, *Clostridium butyricum* and *Lactobacillus acidophilus* endospores on growth performance, nutrient digestibility, meat quality, relative organ weight, microbial shedding and excreta noxious gas emission in broilers. Veterinarni Medicina, 60, 77–86.

[vms3709-bib-0027] Huang, M. K. , Choi, Y. J. , Houde, R. , Lee, J. W. , Lee, B. , & Zhao, X. (2004). Effects of Lactobacilli and an acidophilic fungus on the production performance and immune responses in broiler chickens. Poultry Science, 83, 788–795. 10.1093/ps/83.5.788 15141837

[vms3709-bib-0028] Hussein, A. F. (2014). Effect of biological additives on growth indices and physiological responses of weaned Najdi ram lambs. Journal of Experimental Biology and Agricultural Sciences, 2, 597–607.

[vms3709-bib-0029] Jin, L. Z. , Ho, Y. W. , Abdullah, N. , & Jalaludin, S. (2000). Digestive and bacterial enzyme activities in broilers fed diets supplemented with *Lactobacillus* cultures. Poultry Science, 79, 886–891. 10.1093/ps/79.6.886 10875772

[vms3709-bib-0030] Kazemi, S. A. , Ahmadi, H. , & Torshizi, M. A. K. (2019). Evaluating two multi‐strain probiotics on growth performance, intestinal morphology, lipid oxidation and ileal microflora in chickens. Journal of Animal Physiology and Animal Nutrition, 103, 1399–1407. 10.1111/jpn.13124 31141245

[vms3709-bib-0031] Kumar, S. , Chen, C. , Indugu, N. , Werlang, G. O. , Singh, M. , Kim, W. K. , & Thippareddi, H. (2018) Effect of antibiotic withdrawal in feed on chicken gut microbial dynamics, immunity, growth performance and prevalence of foodborne pathogens. PLoS One, 13(2), e0192450. 10.1371/journal.pone.0192450 29444134 PMC5812630

[vms3709-bib-0032] LaFleur, B. M. , & LaFleur, B. D. (2008). Exploring medical language: A student‐directed approach (7th ed.) St. Louis, MO: Mosby Elsevier.

[vms3709-bib-0033] Lan, P. T. , Binh, L. T. , & Benno, Y. (2003). Impact of two probiotic *Lactobacillus* strains feeding on faecal lactobacilli and weight gains in chicken. Journal of General and Applied Microbiology, 49, 29–36. 10.2323/jgam.49.29 12682864

[vms3709-bib-0034] Liu, X. , Yan, H. , Lv, L. , Xu, Q. , Yin, C. , & Zhang, K. (2012). Growth performance and meat quality of broiler chickens supplemented with *Bacillus licheniformis* in drinking water. Asian–Australs Journal of Animal Science, 25, 682–689. 10.5713/ajas.2011.11334 PMC4093119 25049614

[vms3709-bib-0035] Mahmood, K. , Rahman, S. U. , & Hussai, I. (2014). Non‐antibiotic strategies for the control of necrotic enteritis in poultry. World Poultry Science Journal, 70, 865–879.

[vms3709-bib-0036] Mohan, B. , Kadirvel, R. , Natarajan, M. , & Bhaskaran, M. (1996). Effect of probiotic supplementation on growth, nitrogen utilization and serum cholesterol in broilers. British Poultry Science, 37, 395–401. 10.1080/00071669608417870 8773848

[vms3709-bib-0037] Olnood, C. G. , Beski, S. S. M. , Choct, M. , & Iji, P. A. (2015a). Novel probiotics: Their effects on growth performance, gut development, microbial community and activity of broiler chickens. Animal Nutrition, 1, 184–191. 10.1016/j.aninu.2015.07.003 29767136 PMC5945945

[vms3709-bib-0038] Olnood, C. G. , Beski, S. S. M. , Choct, M. , & Iji, P. A. (2015b). Delivery routes for probiotics: Effects on broiler performance, intestinal morphology and gut microflora. Animal Nutrition, 1, 192–202. 10.1016/j.aninu.2015.07.002 29767168 PMC5945942

[vms3709-bib-0039] Paryad, A. , & Mahmoudi, M. (2008). Effect of different levels of supplemental yeast (*Saccharomyces cerevisiae*) on performance, blood constituents and carcass characteristics of broiler chicks. African Journal of Agricultural Research, 23, 835–842.

[vms3709-bib-0040] Patterson, J. A. , & Burkholder, K. M. (2003). Application of prebiotics and probiotics in poultry production. Poultry Science, 82, 627–631. 10.1093/ps/82.4.627 12710484

[vms3709-bib-0041] Priyankarage, N. , Silva, S. S. P. , Gunaratne, S. P. , Kothalawala, H. , Palliyaguru, M. W. C. D. , & Gunawardana, G. A. (2003). Efficacy of probiotics and their effects on performance, carcase characteristics, intestinal microflora and Salmonella incidence in broilers. Poultry Science, 44, S26–S27.

[vms3709-bib-0042] Rahimi, M. (2009). Effects of probiotic supplementation on performance and humoral immune response of broiler chickens. Book of Proceedings, 2nd Mediterranean Summit of WPSA (pp. 67–69).

[vms3709-bib-0043] Ramlucken, U. , Ramchuran, S. O. , Moonsamy, G. , Lalloo, R. , Thantsha, D. S. , & Rensburg, C. J. (2020). A novel *Bacillus* based multi‐strain probiotic improves growth performance and intestinal properties of *Clostridium perfringens* challenged broilers. Poultry Science, 99, 331–341. 10.3382/ps/pez496 PMC7587899 32416818

[vms3709-bib-0044] Reuben, R. C. , Sarkar, S. L. , Ibnat, H. , Setu, A. A. , Roy, P. C. , & Jahid, I. Q. (2021). Novel multi‐strain probiotics reduces *Pasteurella multocida* induced fowl cholera mortality in broilers. Scientific Reports, 11, 8885. 10.1038/s41598-021-88299-0 33903662 PMC8076301

[vms3709-bib-0045] Reuben, R. C. , Sarkar, S. L. , Roy, P. C. , Rubayet‐Ul‐Alam, A. S. M. , & Jahid, I. K. (2020). Characterization and evaluation of lactic acid bacteria from indigenous raw milk for potential probiotic properties. Journal of Dairy Science, 103(2), 1223–1237. 10.3168/jds.2019-17092 31759592

[vms3709-bib-0046] Reuben, R. C. , Sarkar, S. L. , Roy, P. C. , Rubayet‐Ul‐Alam, A. S. M. , & Jahid, I. K. (2019). Isolation, characterization, and assessment of lactic acid bacteria toward their selection as poultry probiotics. BMC Microbiology, 53, 1–20. 10.1186/s12866-019-1626-0 PMC6852909 31718570

[vms3709-bib-0047] Rocha, T. , Baptista, A. , Donato, T. , Milbradt, E. , Okamoto, A. , & Rodrigues, J. (2012). Evaluation of *in vitro* and *in vivo* adhesion and immunomodulatory effect of *Lactobacillus* species strains isolated from chickens. Poultry Science, 91, 362–369. 10.3382/ps.2011-01803 22252349

[vms3709-bib-0048] Salim, H. , Kang, H. , Akter, N. , Kim, D. , Kim, J. , & Kim, W. K. (2013). Supplementation of direct‐fed microbials as an alternative to antibiotic on growth performance, immune response, cecal microbial population, and ileal morphology of broiler chickens. Poultry Science, 92, 2084–2090. 10.3382/ps.2012-02947 23873556

[vms3709-bib-0049] Samanya, M. , & Yamauchi, K. (2002). Histological alterations of intestinal villi in chickens fed dried *Bacillus subtilis* var. *natto* . Comparative Biochemistry and Physiology, 133, 95–104. 10.1016/s1095-6433(02)00121-6 12160875

[vms3709-bib-0050] Surono, I. S. (2003). *In vitro* probiotic properties of indigenous Dadih lactic acid bacteria. Asian–Australs Journal of Animal Science, 16, 726–731.

[vms3709-bib-0051] Szymanowska‐Powałowska, D. , Orczyk, D. , & Leja, K. (2014). Biotechnological potential of *Clostridium butyricum* bacteria. Brazilian Journal of Microbiology, 45, 892–901. 25477923 10.1590/s1517-83822014000300019 PMC4204974

[vms3709-bib-0052] Teo, A. Y. , & Tan, H. M. (2007). Evaluation of the performance and intestinal gut microflora of broilers fed on corn‐soy diets supplemented with *Bacillus subtilis* PB6 (CloSTAT). Journal of Applied Poultry Research, 16, 296–303.

[vms3709-bib-0053] Timmerman, H. M. , Koning, C. J. , Mulder, L. , Rombouts, F. M. , & Beynen, A. C. (2004). Monostrain, multistrain and multispecies probiotics—A comparison of functionality and efficacy. International Journal of Food Microbiology, 96, 219–233. 15454313 10.1016/j.ijfoodmicro.2004.05.012

[vms3709-bib-0054] Timmerman, H. M. , Mulder, L. , Everts, H. , van Espen, D. C. , van der Wal, E. , & Klaassen, G. (2005). Health and growth of veal calves fed milk replacers with or without probiotics. Journal of Dairy Science, 88, 2154–2165. 15905445 10.3168/jds.S0022-0302(05)72891-5

[vms3709-bib-0055] Van der Wielen, P. W. , Lipman, L. J. A. , van Knapen, F. , & Biesterveld, S. (2002). Competitive exclusion of *Salmonella enterica* serovar enteritidis by *Lactobacillus crispatus* and *Clostridium lactatifermentans* in a sequencing fed‐batch culture. Journal of Applied and Environmental Microbiology, 68, 555–559. 11823190 10.1128/AEM.68.2.555-559.2002 PMC126684

[vms3709-bib-0056] van Dijk, J. E. , Huisman, J. , & Koninkx, J. F. (2002). Structure and functional aspects of a healthy gastrointestinal tract. In: M. C. Blook , H. A. Vahl , L. DeLange , A. E. Vande Braak , G. Hemke (Eds.), Nutrition and health of the gastrointestinal tract (pp. 71–92). Wageningen: Wageningen Academic Publishers.

[vms3709-bib-0057] Yang, C. M. , Cao, G. T. , Ferket, P. R. , Liu, T. T. , Zhou, L. , & Zhang, L. (2012). Effects of probiotic, *Clostridium butyricum*, on growth performance, immune function, and cecal microflora in broiler chickens. Poultry Science, 91, 2121–2129. 10.3382/ps.2011-02131 22912445

[vms3709-bib-0058] Zhang, Z. , & Kim, I. (2014). Effects of multistrain probiotics on growth performance, apparent ileal nutrient digestibility, blood characteristics, cecal microbial shedding, and excreta odor contents in broilers. Poultry Science, 93, 364–370. 10.3382/ps.2013-03314 24570458

[vms3709-bib-0059] Zhang, Z. F. , Cho, J. H. , & Kim, I. H. (2013). Effects of *Bacillus subtilis* UBT‐MO2 on growth performance, relative immune organ weight, gas concentration in excreta, and intestinal microbial shedding in broiler chickens. Livestock Science, 155, 343–347.

