# Supplementary material for: Novel mono‐ and multi‐strain probiotics supplementation modulates growth, intestinal microflora composition and haemato‐biochemical parameters in broiler chickens
Source: Vet Med Sci. 2022 Jan 11;8(2):668–80. doi: 10.1002/vms3.709 (PMC8959300; doi:10.1002/vms3.709)
Supplement: Supplementary file 1 — Supporting Information [file VMS3-8-668-s001.docx]

**Supplementary Table 1**: Ingredient composition and calculated nutrient of basal diet used for the study

| item | starter | finisher |
| --- | --- | --- |
| **Ingredient, g/kg** | |  |
| Wheat (26.00%) | 262.00 | 214.00 |
| Sorghum (15.5%) | 350.25 | 400.00 |
| Mung beans | 100.00 | 100.00 |
| Soybean meal (46%) | 157.00 | 81.50 |
| Limestone | 15.50 | 16.00 |
| Salt | 1.75 | 1.50 |
| Soybean oil | 2.00 | 2.50 |
| Moisture | 8.00 | 6.70 |
| Ash | 7.60 | 6.00 |
| Dicalcium phosphate | 1.50 | 1.50 |
| Lysine | 2.10 | 0.40 |
| Methionine | 2.10 | 1.30 |
| Threonine | 0.20 | 0.15 |
| Vitamin and mineral premix* | 2.50 | 2.50 |
| Crude protein | 200.00 | 190.00 |
| Crude fibre | 35.17 | 43.14 |
| Crude fat | 52.16 | 54.47 |

*Nutrition value per Kg of vitamin and mineral premix contains 140000 IU of vitamin A, 70 mg of vitamin E, 3000 IU of vitamin D3, 4 mg of vitamin K, 3 mg of thiamine, 10 mg of vitaminB2, 8 mg of vitamin B6, 0.04 mg of vitamin B12, 48 mg of niacin, 20 mg of calcium d-pantothenate, 500 mg of choline chloride, 0.20 mg of biotin, 1.8 mg of folic acid, 80 mg of manganese, 70 mg of zinc, 50 mg of iron, 10 mg of copper, 3 mg of iodine, 0.4 mg of selenium, and 0.2 mg of cobalt.

**Supplementary Table 2:** Effects of mono- and multi strains probiotics supplementation pH of GIT of broilers

|  | **Treatment** | |  |  |  |  |  |  | **SEM** | **P-Value** |
| --- | --- | --- | --- | --- | --- | --- | --- | --- | --- | --- |
| **pH** | **NC** | **Ant** | **PC** | **Pa** | **Pp** | **Ef** | **Lp** | **Multi** |  |  |
| **Day 21** |  |  |  |  |  |  |  |  |  |  |
| Gizzard | 3.59^ab^ | 3.12^b^ | 3.78^ab^ | 3.59^ab^ | 4.0^a^ | 3.55^ab^ | 3.19^ab^ | 2.89^c^ | 0.13 | 0.001 |
| Ileum | 5.56^a^ | 5.55^a^ | 5.58^a^ | 5.62^a^ | 5.59^a^ | 5.70^a^ | 5.25^a^ | 5.06^a^ | 0.15 | 0.090 |
| Caecum | 5.97^b^ | 6.30^ab^ | 6.17^ab^ | 6.15^ab^ | 6.50^ab^ | 6.04b^b^ | 6.35b^a^ | 6.65^a^ | 0.08 | 0.001 |
| **Day 35** |  |  |  |  |  |  |  |  |  |  |
| Gizzard | 3.79^ab^ | 3.55^abc^ | 3.63^abc^ | 3.47^bc^ | 3.97^a^ | 3.47^bc^ | 3.42^bc^ | 3.22^c^ | 0.08 | 0.002 |
| Ileum | 5.56^a^ | 5.63^a^ | 5.42^a^ | 5.18^a^ | 5.49^a^ | 5.45^a^ | 5.32^a^ | 5.36^a^ | 0.04 | 0.210 |
| Caecum | 6.31^c^ | 6.78^ab^ | 6.51^bc^ | 7.04^a^ | 6.90^ab^ | 6.89^ab^ | 6.61^bc^ | 6.75^ab^ | 0.08 | 0.015 |

Values are the mean ± standard error of the mean of two replicates. Within each variable, values with the same superscript letter are not significantly different according to Duncan’s multiple range test (P>0.05). NC; Negative control, Ant; Antibiotic supplemented, PC; Positive control, Pa; supplemented with *P. acidilactici* I5, Pp; supplemented with *P. pentosaceus* I13, Ef; supplemented with *E. faecium* C14, Lp; supplemented with *L. plantarum* C16 (Lp), and Multi; supplemented with Multi-strains.
